# Clinical and Molecular Characterization of Pakistani Mucopolysaccharidosis Families with *SGSH* and *GALNS* Deficiencies

**DOI:** 10.3390/genes17040401

**Published:** 2026-03-31

**Authors:** Farheen Nasir Awan, Shumaila Zulfiqar, Liza Eiman, Maria Asif, Muhammad Sajid Hussain, Niklas Dahl, Shahid Mahmood Baig, Hirotsugu Oda

**Affiliations:** 1 Department of Biotechnology, Kinnaird College for Women, Lahore 54000, Pakistan; 2 Cologne Excellence Cluster on Cellular Stress Responses in Aging-Associated Diseases (CECAD), University of Cologne, 50923 Cologne, Germany; 3 Cologne Center for Genomics (CCG), Faculty of Medicine, University Hospital Cologne, University of Cologne, 50923 Cologne, Germany; 4 West German Genome Center (WGGC), University of Cologne, 50923 Cologne, Germany; 5 Department of Immunology, Genetics and Pathology, Uppsala University, 751 85 Uppsala, Sweden; 6 Health Services Academy (HSA), Islamabad 44000, Pakistan; 7 Center for Molecular Medicine Cologne (CMMC), Faculty of Medicine, University Hospital of Cologne, University of Cologne, 50923 Cologne, Germany; 8 Institute for Systemic Inflammation Research (ISEF), University of Lübeck, 23562 Lübeck, Germany

**Keywords:** *SGSH*, *GALNS*, MPS IIIA, MPS IVA, Sanfilippo syndrome, Morquio syndrome, whole-exome sequencing, consanguinity, Mucopolysaccharidoses

## Abstract

**Background:** Mucopolysaccharidoses (MPS) are rare lysosomal storage disorders caused by deficiencies in glycosaminoglycan (GAG)-degrading enzymes, leading to progressive multisystem involvement. **Methods:** We evaluated two unrelated consanguineous Pakistani families, each with three individuals showing features consistent with MPS. Affected individuals in Family 1 presented with developmental regression, severe cognitive impairment, behavioral abnormalities and facial dysmorphisms. The affected individuals in Family 2 showed classical skeletal dysplasia consistent with Morquio syndrome. Whole-exome sequencing (WES), segregation analysis, and in silico protein modeling were performed to identify and characterize pathogenic gene variants. **Results:** Analysis of WES data revealed a homozygous missense variant in the *SGSH* gene [c.548G>A (p.Cys183Tyr)] in the three cases of Family 1 and a homozygous splice-site variant in the *GALNS* gene (c.423-1G>A) in the cases of Family 2. The *SGSH* variant, located within the sulfatase catalytic domain and classified as likely pathogenic (ACMG), is consistent with the Sanfilippo A phenotype and represents the first clinical characterization of this allele. Regarding Family 2, we identified the *GALNS* mutation as a recurrent pathogenic founder allele previously reported in individuals of South Asian descent. Structural modeling of *SGSH* p.Cys183Tyr predicted disruption of a conserved cysteine residue and altered protein stability, likely supporting its deleterious effect. **Conclusions:** This study expands the spectrum of MPS-associated variants in Pakistan. The findings underscore the importance of genomic diagnostics for enabling early detection, accurate classification, and genetic counseling in populations with high consanguinity.

## 1. Introduction

Mucopolysaccharidoses (MPS) are a group of inherited lysosomal storage disorders caused by defects in enzymes responsible for the breakdown of glycosaminoglycans (GAGs) [1,2]. Impaired degradation leads to the progressive accumulation of GAGs within cells and tissues, resulting in multisystem disease that may involve the skeleton, eyes, cardiovascular system, and central nervous system. MPS encompass a wide spectrum of clinical phenotypes, ranging from severe to attenuated forms, and include all major subtypes such as MPS I, II, III, IV, VI, VII and MPS IX which often exhibit overlapping physical features [3,4]. Most MPS types are inherited in an autosomal recessive manner, with the exception of MPS II, which is X-linked [5].

Although individually rare, the combined birth prevalence of MPS is substantial, with reported rates ranging from approximately 1 to 5 per 100,000 live births across different regions [6,7]. Considerable geographic variation exists, largely influenced by founder effects, population structure, and consanguinity. MPS III is more frequently reported in European and Australian cohorts, whereas MPS II predominates in several East Asian populations [8,9,10]. Other forms, such as MPS IVA and MPS VI, show striking regional differences; for example, high rates of MPS VI have been noted in Saudi Arabia and parts of India, while MPS IVA remains uncommon in many Western countries [11,12].

In Pakistan, the true prevalence of MPS remains unknown due to limited diagnostic resources, a lack of systematic epidemiological studies, and the underutilization of genomic testing. However, the high rate of consanguineous marriage estimated at 60% in some communities suggests that autosomal recessive metabolic diseases, including MPS, may be underrecognized [13]. Only a small number of genetically confirmed MPS cases have been reported from the country, including families with MPS I, MPS II, MPS III, and a few with MPS IVA [14,15]. Given this limited data, additional molecular characterization is essential to understand the local mutation spectrum and to guide clinical management.

This study describes six affected individuals from two unrelated consanguineous Pakistani families presenting with features consistent with MPS IIIA (Sanfilippo A syndrome) and MPS IVA (Morquio syndrome). Through whole-exome sequencing segregation analysis, and in silico structural modeling, we identified and characterized a clinically novel pathogenic *SGSH* variant c.548G>A (p.Cys183Tyr) associated with MPS IIIA. Furthermore, we identified a previously reported *GALNS* founder mutation (c.423-1G>A), confirming MPS IVA. Our findings expand current knowledge on the genetics behind MPS in Pakistan and highlight the importance of genomic analysis in populations with high rates of consanguinity.

## 2. Materials and Methods

### 2.1. Family Enrollment and Ethical Approval

Two unrelated consanguineous Pakistani families presenting with syndromic intellectual disability and skeletal abnormalities were recruited. Pedigree analysis indicated autosomal recessive inheritance in both families.

**Figure 1:**
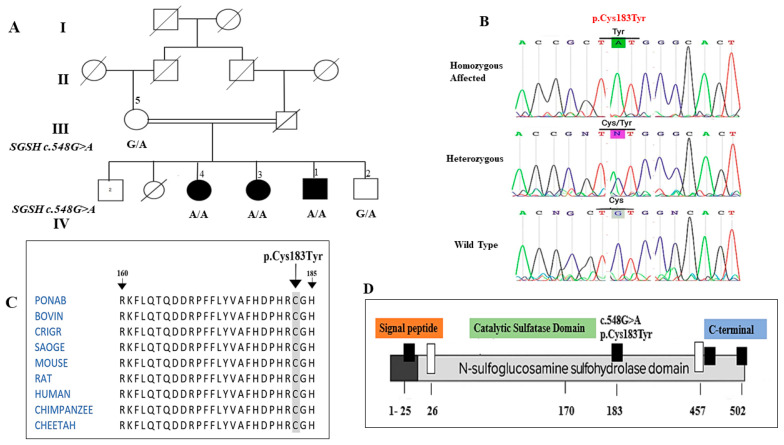
Molecular and structural characterization of the *SGSH* c.548G>A (p.Cys183Tyr) variant in Family 1. (**A**) Pedigree of Family 1 segregating *SGSH* c.548G>A variant. (**B**) Sequence chromatogram of genomic DNA showing part of the SGSH gene obtained from the homozygous affected individuals (IV-1) (**top**), heterozygous mother and sibling (IV:5) (**middle**), and a healthy control (**bottom**). The highlighted region indicates the position of the c.548 G>A transition. (**C**) Degree of conservation of substituted amino acid (Cys183) among vertebrates signifying the functional and structural importance in protein. (**D**) Showing Cys183 mutation in sulfatase catalytic domain of SGSH protein.

**Figure 2:**
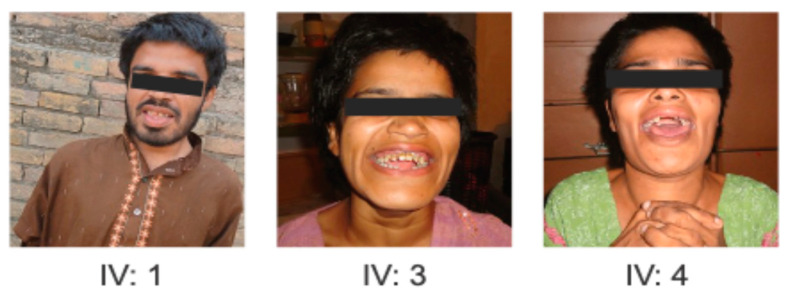
Clinical features of Family 1 patients with Sanfilippo syndrome type A (MPS IIIA) showing characteristic relatively mild facial dysmorphism, coarse facial features and developmental regression.

**Figure 3:**
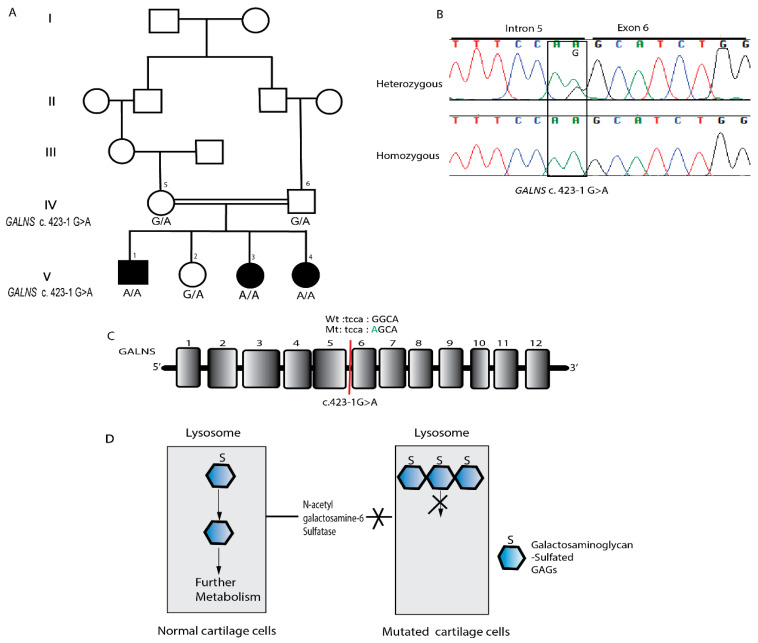
(**A**) Pedigree illustrating autosomal recessive inheritance. (**B**) Sequence chromatogram of *GALNS* showing a segment of exon 6 and intron 5, with a heterozygous individual on top and an affected individual on the bottom. The black box marks the location of the variant. (**C**) Schematic representation of *GALNS* exons highlighting the variant at the exon–intron boundary. (**D**) Illustration of GALNS function in removing sulfate groups from specific glycosaminoglycans (GAGs) during lysosomal degradation. Mutations in GALNS lead to accumulation of sulfated GAGs within lysosomes, disrupting normal protein function and impairing intracellular trafficking.

**Figure 4:**
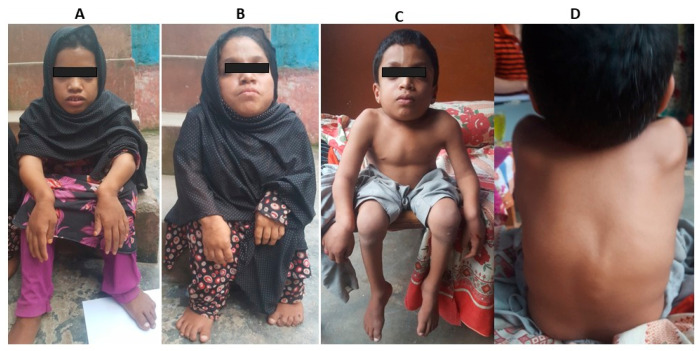
Phenotypic presentations of affected individuals in Family 2. (**A**) Individual V:1 showing facial dysmorphism which include broad mouth, prominent cheekbones, a small and broad nose (**B**) Patient (V:3) showing short legs and arms (**C**) V:4 showing short neck. (**D**) Showing spine abnormality in a patient (V:4).

Family 1 (Rawalpindi, Punjab) included six siblings, three of whom were affected (IV:1, IV:3, IV:4) (Figure 1 and Figure 2). Family 2 (Mansehra, Khyber Pakhtunkhwa) included three affected individuals and three unaffected members (Figure 3 and Figure 4). Neither family had undergone any prior genetic testing. Written informed consent was obtained from all participants or their guardians. Ethical approval was granted by the Research Ethics Committee of National Institute for Biotechnology and Genetic Engineering, Faisalabad (REC NIBGE. No. REC/NIBGE/143/2018) and in accordance with the Declaration of Helsinki [16]. Genomic DNA was extracted using standard protocols.

### 2.2. Clinical Features

In Family 1, three affected siblings (IV:1, IV:3 and IV:4) born to a consanguineous couple. The natural history of the affected siblings in Family 1 followed a classic three-phase progression typical of MPS IIIA. All three individuals had an unremarkable neonatal period and reached early motor milestones (sitting and walking) at appropriate ages. However, a slowing of mental development became apparent between ages 3 and 4 years (Phase 1). This was followed by a period of severe behavioral disturbances, including hyperactivity and aggression, which peaked during late childhood (Phase 2). By their late teens, a significant decline in motor function and the onset of spasticity were observed, leading to their current status of profound cognitive impairment and limited mobility (Phase 3). While progressive neurodegeneration is clinically evident, formal brain MRI imaging and longitudinal neurological scores were unavailable due to the families’ late presentation and geographic displacement. In Family 2, all three affected individuals exhibited stunted growth and short stature. A notably short neck was another consistent feature among them. Spinal abnormalities, specifically kyphoscoliosis, were apparent by 8–10 months of age. Characteristic facial features included a broad mouth, prominent cheekbones, a small and wide nose, and widely spaced eyes (Figure 4A–D). Individuals V:1 and V:3 began walking around the age of five years. All affected members were able to perform self-care, and none experienced seizures or epilepsy. Detailed clinical features of both families have been listed in Table 1. While the skeletal phenotype was highly characteristic of Morquio A syndrome, formal X-ray confirmation was not obtained due to limited access to specialized imaging facilities. Due to regional resource limitations and the geographic displacement of the families, biochemical enzymatic assays and quantitative urinary GAG analysis were not feasible; therefore, diagnosis was established through clinical phenotyping and biallelic molecular findings.

### 2.3. Whole Exome Sequencing and Variant Detection

Whole-exome sequencing (WES) was conducted independently at two international centers. For Family 1, WES of the affected individual IV:1 was performed at the University of Cologne, Germany, and for Family 2 affected individuals V:1 and V:3 were analyzed at Uppsala University, Sweden. Each center processed samples using its validated in-house sequencing and bioinformatics pipelines, as described in detail in previously published protocols [17,18].

Sequencing libraries were prepared using the SureSelect Human All Exon V6 kit (Agilent Technologies, Inc., Santa Clara, CA, USA) and paired-end sequencing was performed on Illumina platforms. Reads were aligned to the GRCh37/hg19 reference genome, and variant calling followed center-specific workflows. Copy-number variation (CNV) assessment was included.

Variant annotation and filtration were performed using internal resources, including the EVAdb database (>20,000 exomes), prioritizing rare homozygous or compound heterozygous variants (minor allele frequency <1%) consistent with recessive inheritance. Variants of interest were classified according to ACMG/AMP guidelines [18,19].

Candidate variants were confirmed, and segregation analysis was performed in all available family members using Sanger sequencing.

### 2.4. In Silico Functional and Structural Analysis

To evaluate the potential functional impact of the identified variants, multiple in silico tools were employed. Pathogenicity predictions were obtained from MutationTaster2, (https://www.mutationtaster.org/), SIFT v6.2.1 (https://sift.bii.a-star.edu.sg/), PROVEAN v1.1.3) (http://provean.jcvi.org), and PolyPhen-2 (http://genetics.bwh.harvard.edu/pph2/). For protein structural analysis, 3D model of SGSH mutant protein were generated using SWISS-MODEL (https://swissmodel.expasy.org/) and Phyre2 v2.0. (https://www.sbg.bio.ic.ac.uk/~phyre2/html/page.cgi?id=index) and visualized with PyMOL v2.5.0 (Schrödinger, LLC) (https://pymol.org/). Additionally, DUET (https://biosig.lab.uq.edu.au/duet/) was used to assess the effect of amino acid substitutions on protein stability through ΔΔG predictions.

## 3. Results

### 3.1. Whole-Exome Sequencing and Variant Identification

In Family 1, WES-analysis identified a homozygous missense variant in *SGSH* (NM_000199.5: c.548G>A; p.Cys183Tyr) in all three affected siblings. The variant lies in exon 5 within the sulfatase catalytic domain (amino acids 26–456), a region essential for enzymatic activity and disulfide bond formation. This represents the first report of this pathogenic variant in the Pakistani population.

In Family 2, WES filtering initially revealed three homozygous candidate variants. Two variants were excluded due to lack of phenotypic relevance. The remaining variant, a previously reported acceptor splice-site alteration in *GALNS* (c.423-1G>A) upstream of exon 6, was considered the most plausible disease-causing variant based on its known association with mucopolysaccharidosis IVA.

### 3.2. Segregation and Sanger Validation

Sanger sequencing confirmed the two gene variants and that each of them segregated with the disease in the respective families, consistent with autosomal recessive inheritance. In Family 1, all affected individuals were homozygous for the *SGSH* p.Cys183Tyr variant, while parents and unaffected siblings were heterozygous carriers (Figure 1B). Conservation analysis (BLASTP, T-Coffee) showed that the Cys183 residue is highly conserved across vertebrates, highlighting its functional importance (Figure 1C).

In Family 2, all affected individuals were homozygous for *GALNS* c.423-1G>A, whereas unaffected members were heterozygous carriers (Figure 3B). This variant is documented in dbSNP and gnomAD and has been previously reported in individuals with MPS IVA.

A summary of the genetic findings for both families is provided in Table 2.

### 3.3. Protein Modeling and Stability Analysis

**Figure 5:**
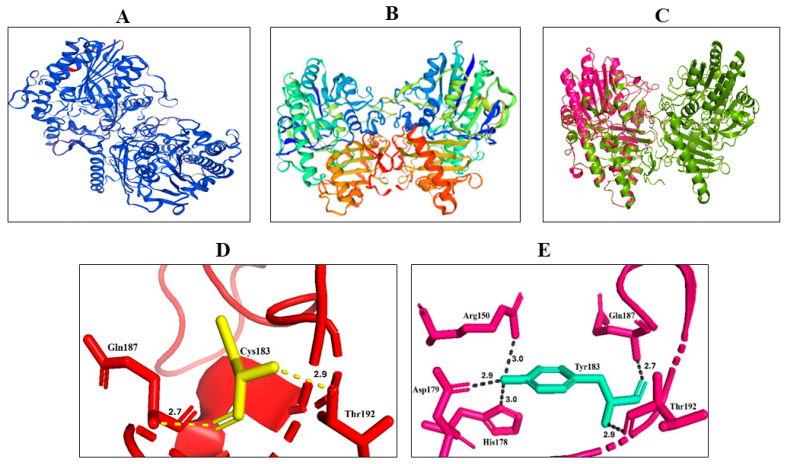
Structural modeling of the SGSH p.Cys183Tyr variant predicts disruption of the catalytic domain. (**A**) Wild type SGSH protein (blue) with the mutation-containing domain highlighted in red predicted by SWISS model. (**B**) Predicted 3D structure of mutant SGSH from SWISS-MODEL. (**C**) Phyre2-predicted mutant SGSH monomer structure (pink) superimposed on the wild type homodimer SGSH structure (green) from PDB. (**D**) Polar bonds of Cys183 in wild type SGSH (**E**) Polar bonds of Tyr183 in mutant SGSH.

To assess the structural consequences of the *SGSH* variant NP_000190.1:p.Cys183Tyr, we modeled the mutant protein using Phyre2 and superimposed the resulting structure onto the wild-type (WT) human *SGSH* homodimer (PDB: 4MHX) (Figure 5A–C). The resulting model demonstrated high reliability with a Global Model Quality Estimate (GMQE) of 0.74 and a QMEANDisCo global score of 0.70 ± 0.05, confirming the structural integrity of the generated coordinates for further analysis. Protein stability analysis using the DUET webserver predicted a ΔΔG of −1.482 kcal/mol for the p.Cys183Tyr substitution. This negative value indicates a significant destabilization of the SGSH protein structure, likely resulting from the loss of the conserved Cys183-Cys194 disulfide bond and the introduction of the bulky tyrosine side chain into the catalytic environment.

While the overall conformation was largely unaltered, there were small structural changes evidenced by the nonalignment of some regions of the wildtype and mutant structures. Consistent with our expectations, Cys183 made polar bonds of 2.7 Å (with Gln187) and 2.9 Å (with Thr192) as shown in Figure 5D, whereas Tyr183 made polar bonds of 3.0 Å (with Arg150), 3.0 Å (with His178), 2.9 Å (with Asp179), 2.7 Å (with Gln187) and 2.9 Å (with Thr192) as shown in (Figure 5E). The critical importance of Cys183 is further supported by the previous structural observation that Cys183 forms a disulfide bond with Cys194, where the Cys183-Cys194 bond stabilizes a long, loop-rich segment (amino acid residues 177–229). Furthermore, Cys183 is located close to the catalytic histidine (His181), which is proposed to act as a proton donor and facilitate the cleavage of the sulfur-nitrogen bond during de-sulfation [20]. Therefore, the substitution of Cys183 by tyrosine would cause a substantial impact on the protein folding.

Domain annotation (STRING) confirmed that Cys183 resides within the N-terminal sulfatase/Phosphodiest domain, consistent with its high evolutionary conservation and established functional relevance. Collectively, the modeling results support a mechanism in which the p.Cys183Tyr variant disrupts a conserved disulfide bond and destabilizes a loop that directly borders the catalytic site. This provides a plausible structural explanation for impaired SGSH activity and is consistent with the clinical severity observed in the affected individuals.

## 4. Discussion

In this study, we investigated two unrelated consanguineous Pakistani families with MPS and identified pathogenic variants in *SGSH* (MPS IIIA) and *GALNS* (MPS IVA), expanding the molecular spectrum of MPS in this population.

In Family 1, a homozygous missense variant in *SGSH* (NM_000199.5:c.548G>A; p.Cys183Tyr; rs1329133410) was identified in three affected siblings. Although this variant is documented in public databases, it has not yet been reported in any peer-reviewed publications. According to ACMG guidelines, this variant is classified as likely pathogenic (PM2, PP3, PP4, PP1) based on its location in the functional sulfatase catalytic domain, absence in population databases, predicted deleterious effect on protein structure, conservation across species, and segregation in affected family members (Table 3) (Figure 1). Clinically, the affected individuals presented with early-onset global developmental delay, profound intellectual disability, minimal speech, and behavioral disturbances consistent with Sanfilippo syndrome type A (MPS IIIA) [21,22,23]. Sanfilippo syndrome, also known as mucopolysaccharidosis (MPS) type III, is one of five autosomal recessive, neurodegenerative lysosomal storage disorders. Its clinical manifestations result from the incomplete lysosomal breakdown of heparan sulfate [24]. In silico analyses predicted destabilization of the protein, disruption of the catalytic domain, loss of a critical disulfide bond, and altered polar interactions, supporting its deleterious effect on enzymatic function.

In Family 2, a homozygous splice-site variant in *GALNS* (c.423-1G>A) was segregating with Morquio syndrome (MPS IVA). To date, this variant has been reported in a single case from Afghanistan [25], but its recurrence in regional populations highlights the importance of population-specific genetic screening for MPS IVA. Clinically, three affected individuals in this family exhibited characteristic skeletal dysplasia, short stature, and motor delays consistent with previously reported MPS IVA phenotypes [26,27]. The variant predicted altered splicing of *GALNS* mRNA and a skipping of exon 6 resulting in a connection of exon 5 directly to exon 7. A RT-PCR analysis of *GALNS* mRNA spanning exon 6 produced a smaller-than-expected product in an affected family member when compared to a control, confirming complete skipping of exon 6 [25]. The clinical features of the affected individuals in Family 2 are consistent with those reported in previous MPS IVA cases. The two families demonstrate the broad phenotypic spectrum of MPS and their genotype–phenotype correlations. While *SGSH* variants primarily affect the central nervous system, *GALNS* variants predominantly impact skeletal development [28,29].

In regions with high rates of consanguinity, molecular confirmation is essential for precise genetic counseling, enabling carrier screening for at-risk relatives and providing the foundation for prenatal or preimplantation genetic diagnosis (PGD). Furthermore, establishing a molecular diagnosis provides a critical baseline for potential inclusion in emerging therapeutic trials, such as substrate reduction therapy for MPS IIIA or enzyme replacement therapy (ERT) for MPS IVA. Although clinical management for the affected individuals in this study remains largely supportive, early molecular diagnosis of Morquio syndrome is increasingly relevant due to the availability of ERTs like elosulfase alfa, which has been shown to improve functional capacity and growth in pediatric cohorts when initiated before the onset of irreversible clinical damage. Integrating these genomic findings into routine practice can therefore facilitate more proactive clinical interventions and informed reproductive choices for affected families [30,31].

Whole-exome sequencing (WES) facilitated accurate molecular diagnosis, allowing for precise identification of pathogenic variants, carrier detection, and informed genetic counseling [32,33]. These results demonstrate the value of integrating clinical assessment with genomic and in silico analyses, particularly for rare or novel variants in underrepresented populations.

These findings expand the genetic and clinical landscape of MPS in Pakistan and emphasize the importance of early molecular diagnosis for effective management, genetic counseling, and potential inclusion in future therapeutic trials.

### Limitations of the Study

Despite providing novel insights into MPS in Pakistan, this study has several limitations. First, the small sample size, comprising only two families with six affected individuals, limits the generalizability of the findings to the broader population. This also reflects the scarcity of population-based data in Pakistan, where the true prevalence and carrier frequency of these variants remain unknown. Furthermore, it should be noted that functional validation of the identified *SGSH* variant was not performed; predictions of pathogenicity were based on in silico analyses and structural modeling.

## 5. Conclusions

This study identifies a previously unreported likely pathogenic *SGSH* variant consistent with MPS IIIA in three members of a Pakistani family and confirms a recurrent *GALNS* splice-site variant associated with MPS IVA in a second family. These findings broaden the known spectrum of MPS-related variants in Pakistan and underscore the importance of genomic diagnostics in populations with high rates of consanguinity. Early molecular confirmation can support tailored clinical management, enable precise genetic counseling, and contribute to future national data on rare hereditary disorders.

## Figures and Tables

**Table 1:** Clinical features of individuals with homozygous *SGSH* and *GALNS* gene variants, respectively.

Clinical Feature	Family 1—SGSH (MPS IIIA)	Family 2—GALNS (MPS IVA)
Individuals	IV:1, IV:3, IV:4	V:1, V:3, V:4
Age at Examination	23–28 years	5–12 years
Natural History	Phase 1: Normal early milestones. Phase 2: Cognitive/behavioral decline (~4 years). Phase 3: Severe motor decline/spasticity (late teens).	Infancy: Onset of kyphoscoliosis (~8–10 months). Early Childhood: Delayed walking (~5 years) due to skeletal dysplasia.
Onset	Early childhood	Congenital/early infancy
Growth Parameters	Normal	Short-trunk dwarfism; severe growth delay
Intellectual Status	Severe to profound disability	Normal intellect
Speech and Language	Minimal speech; poor comprehension	Age-appropriate speech
Behavioral Features	Aggression, hyperactivity, wandering	None were significant
Facial Features	Relatively mild facial coarsening; broad nasal bridge	Broad mouth, prominent cheekbones, wide-set eyes
Skeletal Findings	Mild spasticity; no major deformities	Dysostosis multiplex (Kyphoscoliosis, hip dysplasia, pectus carinatum)
Tone/Movement	Spasticity of hands and feet	Joint laxity; no spasticity
Audiometry/Hearing	Not performed	Not performed
Echocardiography	Not performed	Not performed
Ocular (Slit-lamp)	No corneal clouding (Visual inspection); Slit-lamp not performed	No corneal clouding (Visual inspection); Slit-lamp not performed
Abdominal Ultrasound	No hepatosplenomegaly reported	No hepatosplenomegaly reported
Radiology (X-rays)	Not performed	Not performed; pathognomonic physical features of dysostosis multiplex (e.g., pectus carinatum and kyphoscoliosis) were identified via clinical examination. Formal radiography was unavailable due to regional resource limitations and geographic constraints.
Current Status	Bed-bound or severely limited mobility	Ambulatory with significant gait abnormalities

**Table 2:** Genetic characterization of variants in the *SGSH* and *GALNS* genes.

Feature	Family 1—*SGSH* (MPS IIIA Associated) c.548G>A; p.Cys183Tyr	Family 2—*GALNS* (MPS IVA Associated) c.423-1G>A (Splice-Site Variant)
Variant identified	SGSH c.548G>A (p.Cys183Tyr), homozygous	GALNS c.423-1G>A (splice-site), homozygous
Segregation	All affected siblings homozygous; parents and unaffected members heterozygous	All affected individuals homozygous; parents and unaffected members heterozygous
Mutation type	Missense	Splice-site
Conservation	Cys183 highly conserved across vertebrates	N/A (splice-site)
MutationTaster	0.999 (disease-causing)	0.9 (disease-causing)
SIFT	0.00 (not tolerated)	N/A
PROVEAN	–8.901 (deleterious)	N/A
PolyPhen-2	0.918 (possibly damaging)	1 (probably damaging)
Structural impact	Disruption of catalytic domain, loss of disulfide bond, altered polar interactions	Likely affects splicing → abnormal protein or loss of function

N/A: Not Applicable; SIFT: Sorting Intolerant From Tolerant; PROVEAN: Protein Variation Effect Analyzer.

**Table 3:** ACMG/AMP pathogenicity classification of identified variants.

Variant (HGVS)	Criteria	Evidence/Justification	Strength
SGSH (NM_000199.5)	PM2	Absent from control populations (gnomAD, ExAC, 1000 Genomes).	Moderate
c.548G>A	PP3	Multiple in silico tools (PolyPhen-2, SIFT, REVEL) predict a deleterious effect on the protein.	Supporting
p.Cys183Tyr	PP1	Co-segregation with disease in three affected siblings within a consanguineous family.	Supporting
	PP4	Patient phenotype (developmental regression, mild coarsening) is highly specific for MPS IIIA.	Supporting
Classification		Likely Pathogenic (Total: 1 Moderate + 3 Supporting).	
GALNS (NM_000512.5)	PVS1	Splice-site mutation (canonical -1 position) predicted to cause exon 6 skipping/loss of function.	Very Strong
c.423-1G>A	PM2	Extremely rare in global population databases.	Moderate
(Splice site)	PP4	Clinical features (skeletal dysplasia, short stature) are pathognomonic for MPS IVA.	Supporting
	PS4	Previously reported as a founder mutation in the Pakistani population.	Strong
Classification		Pathogenic (Total: 1 Very Strong + 1 Strong + 1 Moderate + 1 Supporting).	

HGVS: Human Genome Variation Society; gnomAD: Genome Aggregation Database; ExAC: Exome Aggregation Consortium; SIFT: Sorting Intolerant From Tolerant; REVEL: Rare Exome Variant Ensemble Learner; MPS: Mucopolysaccharidosis.

## Data Availability

The data presented in this study are available on request from the corresponding author.

## Abbreviations

ACMG	American College of Medical Genetics and Genomics
CMMC	Center for Molecular Medicine Cologne
GAGs	Glycosaminoglycans
GALNS	Galactosamine (N-acetyl)-6-sulfatase
HS	Heparan sulfate
KS	Keratan sulfate
MAF	Minor allele frequency
MPS	Mucopolysaccharidosis
MPS IIIA	Mucopolysaccharidosis Type IIIA (Sanfilippo A syndrome)
MPS IVA	Mucopolysaccharidosis Type IVA (Morquio A syndrome)
NCBI	National Center for Biotechnology Information
PolyPhen-2	Polymorphism Phenotyping v2
SGSH	N-sulfoglucosamine sulfohydrolase
SIFT	Sorting Intolerant From Tolerant
VUS	Variant of uncertain significance
WES	Whole-exome sequencing
